# Cultivating a more effective culture to advance the engineering of microbial communities

**DOI:** 10.1098/rsfs.2022.0073

**Published:** 2023-02-10

**Authors:** S. Jane Fowler, Thomas P. Curtis

**Affiliations:** ^1^ Department of Biology, Simon Fraser University, Burnaby, Canada; ^2^ School of Engineering, Newcastle University, Newcastle upon Tyne, UK

**Keywords:** culture, academia, collaboration, microbial ecology, environmental engineering, leadership

This theme issue holds contributions from a diverse group of individuals and research groups all dedicated to applying the power and principles of microbial ecology to create the environmental biotechnologies needed in the twenty-first century. These people came together in March 2022 at a Royal Society Theo Murphy meeting to discuss the matter.

This is a vibrant field with many opportunities, challenges and barriers. In the final session of the meeting, we had participants break into small groups and asked them to discuss how we could accelerate progress. Progress to develop the new technologies that would help us to solve some of the grand challenges that humanity currently faces.

To our surprise, every single group identified the culture of academia as a key issue impeding progress. We learnt that our culture prevents successful cross-disciplinary collaboration. We learnt that the competitive nature of research environments and the lack of inclusivity make us less than the sum of our parts. We heard how the reward structure of academia perversely incentivizes those activities and behaviours that hamper successful trans-disciplinary collaboration. Of course, such a diverse group brought a variety of experiences to the discussion. Some were fortunate to have experienced supportive, collegiate and creative cultures. Others less so. Nevertheless, even the most fortunate of us were touched by the unpleasant consequences of the pervasive rules of the academic game.

In order to move faster and more effectively as a field, we need to build a new culture that is focused on collaboration and better solutions rather than one that is centred on competition and metrics.

What then is our culture? It is simply the values, norms and behaviours that we espouse as a community. The culture of microbial ecologists and engineering biologists naturally reflects those of our societies and the demands and rewards of our employers and employment, and thus much of twenty-first century science. However, there is no reason to assume or accept that the culture that spontaneously arises is the culture we want. Indeed our colleagues have made it abundantly clear that we do not have the culture we need or desire.

We envision a culture that allows all of us to have fulfilling and enriched research careers and to meet the very real societal challenges that we face. The clarion call from our confederates is a clear confirmation that we need to do better in both the quality and the effectiveness of our research environments. We argue that the two are intimately connected; an improved research culture will not simply bring us more fulfilling careers, but also more effective ones. Perhaps one of the most pernicious ideas in contemporary academia is that an unpleasant culture is somehow more effective at creating progress and societal solutions. We contest that tacit assumption.

Recognizing and acknowledging that a cultural change is needed is the first step to change. That being said, people have been talking about the ineffective and often unpleasant culture in academia for some time. Positive change, or calls for change, have come most recently from the importance of equity, diversity and inclusion (EDI) being recognized across academia. This is slowly leading to changes at all levels from research groups to funding bodies and has expanded the discourse on issues of culture.

In order to institute permanent changes, a critical mass is needed [1]. Have we reached this critical mass? We would argue that the widespread recognition of a pervasive cultural problem in this workshop suggests that we may well have. To build a new culture, we must define a new ethos upon which to operate—the values and principles that will define our attitudes and behaviours going forward. We have identified the following values upon which a more effective and inclusive culture can be built.

*Collaboration and teamwork*—We can all be more successful when we work together and support each other. It is not possible for any one person to know or be good at everything, so to make progress in the grand challenges we face, we must work collectively. The competitive and individualized culture that pervades academia is unnecessary and is so often detrimental to progress, while being beneficial to the careers of a select few. Competition and recognition of individuals rather than groups remains the basis for so many rewards in academia including grant funding, professional advancement and awards. However, the high level of specialization of individual researchers or research groups means that none of us are equipped on our own to solve the grand challenges facing humanity. Collaboration is not a nice idea. It is a necessity.

*Effective communication*—Especially listening, while putting aside one's own opinions and biases. Many of us are terrible (or at least highly selective) listeners. We have learned to optimize what we pay attention to because there is so much to do, but this does not always serve us well. We also must learn to communicate across disciplines and across sectors and not only to the narrow range of people who speak our language.

*Respect and humility*—Recognize one another's value and treat each other with kindness and consideration, regardless of seniority, gender or any factor that society has traditionally used to keep their in-groups small and homogeneous. This includes recognizing the limitations of your own knowledge and seeking experts in other areas to provide necessary expertise. It also requires giving and being receptive to respectful feedback and recognizing and celebrating your own and each other's successes.

*Integrity*—Academic honesty, respect for your colleagues and their contributions. The recent scandal in Alzheimer's disease research perfectly illustrates how dishonesty can result in billions of dollars of research funding wasted, and the unnecessary loss of many years that may have resulted in untold unnecessary deaths [2]. Although this is an egregious example, less harmful but still detrimental examples of not recognizing the contributions of others, and obstinate pursuit of old and sometimes discredited ideas abound in academia.

*Innovation and boldness*—These are more likely in an environment where you feel supported and recognized, in which you can take the risks to do things that may be transformational. This also supports a collective culture of scientific failures and success rather than the hierarchical and individualized approach that is often prevalent in our institutions.

*Leadership*—Cultural shifts require committed leadership to inspire change and, on occasion, to confront and persuade those wedded to the old ways. These leaders must wholeheartedly adopt the new framework and embrace their role and dedication to this change. This will, at certain junctures, demand personal sacrifice for collective benefit. Although confronting the old ways head-on and making sometimes uncomfortable decisions will initially be a difficult role to take on, it will be easier to achieve with the critical mass whom we know are inspired to enact this vision. Once the culture becomes the new norm, leading in this way will become easier and more comfortable.

Importantly, these changes must occur at all levels to be truly effective in changing the culture. If funding bodies and scientific societies continue to award their greatest accolades to individuals that benefit from the ‘old academia’ then little progress will be made. As an example, while changes to grant applications that require outlining the approaches used to ensure an equitable and inclusive working environment force people to think about these issues, if they are not eventually followed up on and enforced, then they will amount to naught. They are merely boxes to tick while the old culture continues to thrive without consequence. Furthermore, if the greatest rewards that science offers are at the individual level, the trans-disciplinary collaborations that could provide transformational solutions will struggle to persist.

*Mission based research*—We speculate that the adoption of a mission-based approach might engender the collective and collaborative mindset. Missions can be a vehicle for our culture and our science. They could easily be adopted at the level of scientific societies or funding agencies, or on smaller scales within universities or groups of researchers [3].

What is a mission? A mission is a project with a specific goal. The Apollo programme was the archetypal mission-based programme. In a mission you agree on a goal and a strategy, and you work together, and continue to work together, to attain that goal. A mission has certain characteristics. It should be consequential: contribute to our societal goals. It should be achievable, at least in principle. It should have an agreed upon strategy, but not necessarily agreed upon ‘tactics’. Success means success in the mission. If the mission succeeds, we have all succeeded. If a mission fails, we have all failed. However, individual failures can still contribute to collective success. This approach has been strongly advocated by Mariana Mazzucato as an antidote to the laissez-faire approach to innovation. It may also be a solution to the laissez-faire approach to research in engineering biology. A successful mission requires the collaborative culture to which we aspire and will deliver the solutions we believe we are capable of and that our societies need and, increasingly, demand. There is no shortage of missions.

The synthesis of microbial ecology and engineering holds enormous potential for solving many of the grand challenges of the twenty-first century. We are grateful for the insight and honesty of our colleagues who have shown us that the path towards unleashing this potential lies not through a better bacterium, computer, or sequencer but a better appreciation of and regard for our peers and trainees.

It is apt that our community should raise this challenge. For the power of an engineered microbial community is not the product of a single species, much less a single individual microbe. The power emerges from myriad interactions between countless species with wondrous properties when they are placed in the right environment. Our scientific and engineering power likewise emerges through myriad interactions between uncounted individuals with marvellous properties. That power lies in us collectively, not in one brilliant individual. We, too, just need the right environment.

## Data Availability

This article has no additional data.

## References

[1] Centola D, Becker J, Brackbill D, Baronchelli A. 2018 Experimental evidence for tipping points in social convention. Science **360**, 1116-1119. (10.1126/science.aas8827) 29880688

[2] Piller C. 2022 Blots on a field? Science **377**, 358-363. (10.1126/science.add9993) 35862524

[3] Mazzucato M. 2018 *Mission-oriented research & innovation in the European Union: a problem-solving approach to fuel innovation-led growth*. European Commission. (10.2777/360325)

